# Cases of spontaneous reattachment of rhegmatogenous retinal detachment

**DOI:** 10.1093/omcr/omab076

**Published:** 2021-09-13

**Authors:** Lukpan Orazbekov, Kamilya Zhanbolat, Kairat Ruslanuly

**Affiliations:** 1 First Ophthalmology Department, Kazakh Eye Research Institute, Almaty, Kazakhstan; 2 Postgraduate Education Department, Kazakh Eye Research Institute, Almaty, Kazakhstan

## Abstract

Report on clinical cases of spontaneous reattachment of rhegmatogenous retinal detachment (RRD). From 2014 to 2020 we diagnosed four patients with spontaneous reattachment of RRD. We conducted a review of the relevant medical records, focusing on the initial symptoms at presentation, the initial diagnoses, with a further observation of the patients next 3 years. The patients were re-examined 3 years later after cases of spontaneous reattachment of RRD. Three years after a case of spontaneous reattachment of RRD during reexamination in four patients (four eyes), redetachment was not detected. The mentioned clinical cases indicate the possibility of finding new approaches to RRD management.

## INTRODUCTION

Rhegmatogenous retinal detachment (RRD) is a progressive state requiring surgical intervention aimed to block retinal tears (RT) and remove vitreoretinal traction. It is known that surgery is the sole management of RRD and must be performed immediately. Scleral buckling and vitrectomy are targeted to block RT and reduce vitreoretinal traction with retinal reattachment as a potential outcome. As far as literature review shows, undiagnosed RRD or delay in surgery may result with spontaneous reattachment of rhegmatogenous retinal detachment (SRRRD) [1].

## CASE REPORT

### Case No. 1

**Figure 1:**
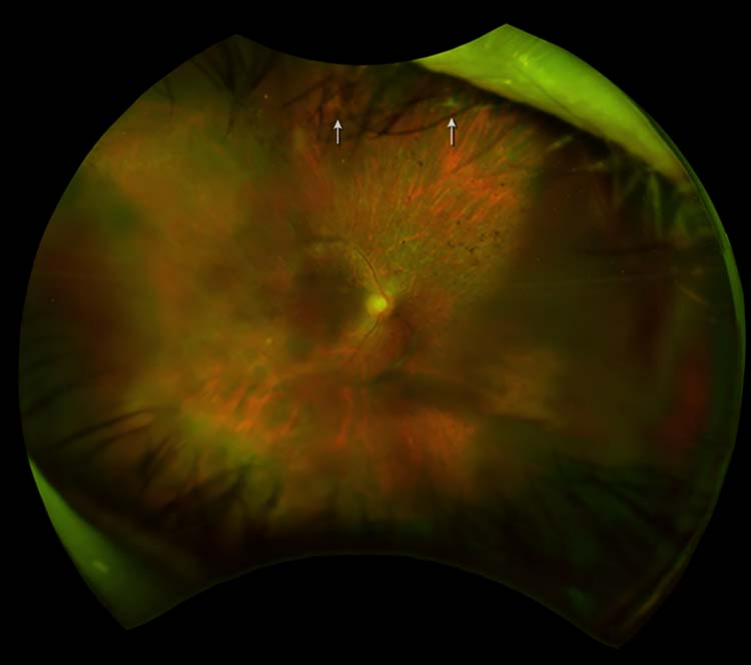
Right eye fundus of Case 1 after SRRRD. Optos ultrawidefield scanning laser ophthalmoscope (SLO) image (Daytona, Optos, Dunfermline, Scotland, UK): The retinal tears at 12:00 and 1:00 o’clock (arrowheads).

A 45-year-old female noted a sudden loss of vision on the right eye (OD) a month ago. The best-corrected visual acuity (BCVA) was incorrect light projection OD and 20/200 in the left eye (OS); low visual acuity OS caused by myopic maculopathy. Dilated fundus examination (DFE) OD revealed posterior vitreous detachment (PVD) and RRD in three quadrants with retinal folds and RT at 12:00 and 1:00 o’clock at the periphery. Vitreoretinal surgery was offered to the patient. One month later, SRRRD was diagnosed (Fig. 1) and retinal laser coagulation (RLC) was performed around RT. One week later, BCVA OD was correct light projection; another week later, it became 20/4000. A year later, cataract surgery was performed and BCVA OD reached 20/800.

### Case No. 2

**Figure 2:**
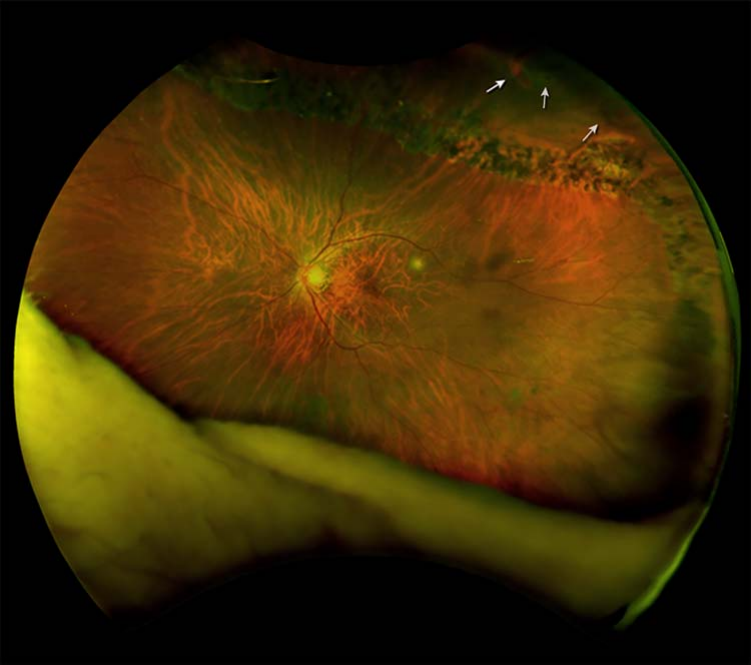
Left eye fundus of Case 2 after SRRRD. (A) SLO image: Laser spots over the lattice degeneration from 10:30 to 3:00 o’clock. U-shaped retinal tear at 1.00 o’clock, the retinal tears at 1:30 and 2:00 o’clock (arrowheads).

A 69-year-old female noticed flashes and a ‘curtain’ in front of both eyes (OU). BCVA was correct light projection OD and 20/40 OS. DFE OU revealed RRD: OD revealed PVD and RRD from 10:00 to 5:00 o’clock; OS revealed PVD and RRD in two quadrants with RT from 10:30 to 3:00 o’clock. One month later, at the time of admission for vitreoretinal surgery for RRD OD, SRRRD OS was diagnosed (Fig. 2); RLC was performed around the lattice degeneration and peripheral RT. One month later, BCVA OS was 20/30; a year after, it was 20/25.

### Case No. 3

**Figure 3:**
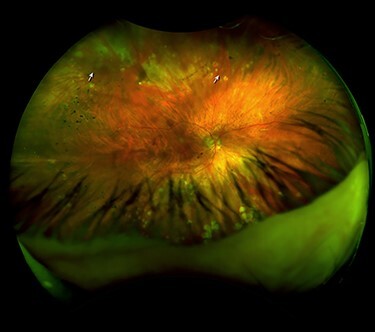
Right eye fundus of Case 3 after SRRRD. (A) SLO image: Old laser spots on the periphery and equator at 360°. The atrophic holes at 1:00 and 11.00 o’clock (arrowheads).

A 56-year-old male noted a loss of vision and flashes OD. BCVA was 20/400 OD and 40/200 OS; low visual acuity OS caused by myopic maculopathy. DFE OD revealed attached posterior vitreous with RRD in three quadrants involving the macula with atrophic retinal holes at 1:00 and 11:00 o’clock, the retina had fibrotic changes with pigment deposits on its surface. Vitreoretinal surgery was offered to the patient, but one or a month later SRRRD was diagnosed (Fig. 3) and RLC was performed over the atrophic retinal holes at 11:00 and 1:00 o’clock. One week later, BCVA OD was 20/100; another month later, it became 20/60. A year later, cataract surgery was performed and BCVA OD reached 20/30.

### Case No. 4

**Figure 4:**
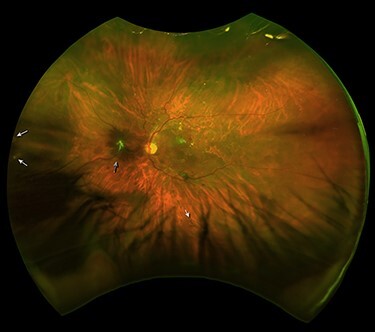
Left eye fundus of Case 4 after SRRRD. (A) SLO image: Glial tissue left to the optic disc (black arrowhead). The retinal tears at 9:00 and 6:00 o’clock (arrowheads).

A 59-year-old male noted a decrease in vision OS. The patient had diabetic retinopathy, a proliferative stage with maculopathy. BCVA was 20/4000 OS and 20/50 OD; low visual acuity OD caused by diabetic macular edema. DFE OS revealed PVD with RRD in two upper quadrants involving the macula with RT at 12:30, 6:00 and 9:00 o’clock. Vitreoretinal surgery was offered to the patient. A month later, SRRRD was diagnosed (Fig. 4) and RLC over RT was performed. DFE OS revealed macular edema, multiple microaneurysms and microhemorrhages, hard and soft exudates, neovascularization, laser spots on periphery. BCVA OS was 20/100.

Individual patient data are summarized in Table 1. The initial diagnoses of all patients at time of referral were RRD. After confirmation of RRD, surgery was offered; delay in surgery was due to the circumstances beyond our control (patient reluctance, problem of insurance coverage). In all cases, SRRRD was diagnosed a month later; there was no retinal redetachment over a 3 years follow-up period and BCVA remained stable.

**Table 1:** Patients’ data

Case №	Gender	Age	Size of the eye, mm	Refraction (spherical equivalent diopters)	Visual acuity (before SRRRD)	Visual acuity (after SRRRD)	Visual acuity (3 years after SRR)	Retinal detachment area	PVD
1 (OD)	F	45	22.41	−6.25	Pr.l.incertae	20/4000	20/800	3 quadrants	+
2 (OS)	F	69	24.53	−3.5	20/40	20/30	20/25	2 quadrants	+
3 (OD)	M	56	24.26	−5.5	20/400	20/60	20/30	3 quadrants	−
4 (OS)	M	59	23.16	−0.75	20/4000	20/100	20/100	2 quadrants	+

## DISCUSSION

Algvere et al. [2] and Lean et al. [3] have suggested that SRRRD may occur after immobilization of an eye. However, they pointed out that detached retina with vitreous traction and fixed retinal folds showed an insignificant tendency toward spontaneous reattachment.

De Jong et al. [4] concluded that limiting head and eye movements is enough to prevent RRD progression, but it should not be counted as an only contributing factor to SRRRD; the authors supposed that the gravitational force effect on the retina and subretinal fluid is small because of the no density difference betweenthem.

Some studies have suggested that the basis for the development of SRRRD presumably involves the occurrence of a complete PVD that in most cases relieves vitreoretinal traction [5, 6]. Inflammatory response to RT in addition to RRD triggers microglial and Müller cells migration and their massive proliferation at the vitreoretinal interface to establish a glial scar that closes RT and, in our opinion, facilitates developing SRRRD [7, 8]. Chung et al. [1] suggested that, after a partial PVD that relieves vitreoretinal traction, vitreous fibers running parallel to the retinal plane may close RT and interrupt the fluid inflow; in another words, the occurrence of PVD with a consequent RT plugging; subsequently, RPE removes residual subretinal fluid by active transport and/or by Starling force (the balance between capillary pressure, interstitial pressure, and osmotic pressure) that is conducive to SRRRD [9].

In our study patients were not limited in head and eye movement or neither in a bed position, they had various types of RT, one patient had proliferative diabetic retinopathy, one patient had no PVD; the clinical variability of the SRRRD may contradicts studies above.

There were several limitations in our study. First, the limited number of the cases and retrospective nature of this study. Second, all patients were not comprehensively examined by imaging tools with focus imaging of RT and detachment before SRRRD to determine the pathogenesis of SRRRD. We recognize and do not undermine the importance of vitreoretinal surgery in RRD; we strongly recommended surgery to all our patients.

To summarize, SRRRD is a rare event in ophthalmologist’s practice, accumulating case reports may provide significant information in understanding the current phenomenon. In certain cases, persistent reattachment of the retina after RRD without any intervention is possible. The mentioned clinical cases indicate the possibility of finding new approaches to RRD management.
